# On Pultaceous Chancre

**Published:** 1855-12

**Authors:** 


On Pultaceous Chancre.—M. Robert observes that pseudo-membraneous or diphtheric chancre is found in persons of quite opposite temperament and constitution. Some are robust, offering all the attributes of the sanguineous temperament, while others are weak and lymphatic, and of a chlorotic color. In the first of these, the circumference of the chancre is red, and presents here and there turgescent prominences, from which, on the least touch, arterial-like blood gushes forth. In the others the sore is flabby, exhiting only a few vesicular granulations, from which pale blood, as in the anæmic, escapes. In both, however, there is deposited a more or less thick and adherent pseudo-membrane, composed of the same elements, and soluble in ammonia. The deposition of this plastic substance may be explained by the supposition that there is a change in the normal proportions of the fibrin and globules of the blood. In the feeble such excess is relative, from a diminution in the quantity of globules, while in the robust the quantity of fibrin becomes absolutely greater.

Treatment must be directed against the radical cause of the deposition, and not to mere local effects. M. Robert has almost always found these membranes reproduced when their removal has been accomplished by caustic or other local means. The indication is to re-establish the equilibrium between the elements of the blood, and, if we only administer iron and tonics indiscriminately, although in one class of cases we may do great good, in the other we shall induce inflammatory gangrene. The excess of fibrin is best influenced by the employment of bicarbonate of soda, which may be given in doses of ℥jss. per diem, the sore being dressed with a solution, containing ten per cent.—(*L'Union Méd.*) *Ibid.*

